# Sex and Gender Differences in Patients with Gastric Cancer: A Systematic Review

**DOI:** 10.3390/jcm15124788

**Published:** 2026-06-19

**Authors:** Nerea Escandell Marí, Marta Sánchez-Ric, Marina Velez, Sabela Carballal, Leticia Moreira

**Affiliations:** 1 Facultat de Medicina i Ciències de la Salut, Universitat de Barcelona (UB), c. Casanova, 143, 08036 Barcelona, Spain; 2 Servei de Gastroenterologia, Hospital Clínic de Barcelona, Universitat de Barcelona (UB), c. Villarroel, 170, 08036 Barcelona, Spain; 3 Institut d’Investigacions Biomèdiques August Pi i Sunyer (IDIBAPS), c. Villarroel, 170, 08036 Barcelona, Spain; 4 Centro de Investigación Biomédica en Red de Enfermedades Hepáticas y Digestivas (CIBEREHD), c. Monforte de Lemos, 3-5, 28029 Madrid, Spain

**Keywords:** gastric cancer, sex differences, gender differences, risk factors, survival

## Abstract

**Background:** Gastric cancer is a major global health concern. Although sex- and gender-based differences have been described, they are not yet well established, and the available evidence is often inconsistent. This systematic review aims to explore these differences in the incidence, clinicopathological characteristics, risk factors, treatment, and survival of gastric cancer, thereby contributing to healthcare equity. **Methods:** A systematic search was conducted in the main medical bibliographic databases (PubMed, Embase and Web of Science) in February 2026 following the PRISMA 2020 guidelines. Studies on gastric cancer were selected based on predefined inclusion and exclusion criteria. The results were synthesized qualitatively according to incidence, clinicopathological characteristics, risk factors, treatment outcomes, and survival. Due to the heterogeneity and predominantly observational design of the included studies, no meta-analysis or formal risk-of-bias assessment was conducted. **Results:** A total of 38 studies, involving more than 500,000 participants, were included. Most reported a higher incidence of gastric cancer in men, with a predominance of intestinal and well-differentiated tumors, while diffuse and poorly differentiated tumors were more common in women. Men showed higher rates of smoking, alcohol consumption, and postoperative complications. Overall survival tended to be higher in women, especially in early stages, although some studies described worse outcomes among young women. **Conclusions:** This review highlights relevant sex- and gender-related differences in gastric cancer and underscores the need to systematically incorporate these variables into future research to advance towards more personalized medicine. The available evidence was limited by the predominance of retrospective observational studies and heterogeneity across study designs and reported outcomes.

## 1. Introduction

Gastric cancer remains a significant global health concern, accounting for a substantial burden of morbidity and mortality worldwide. According to the GLOBOCAN estimates, there were over 968,000 new cases in 2022 and close to 660,000 deaths [1]. It is the fifth most common cancer in terms of incidence and mortality, following lung, colorectal, liver, and breast cancers [2]. The highest incidence rates are observed in East Asia, Eastern Europe, and South America. However, there has been a decline in incidence and mortality rates since the mid-20th century, mainly due to improved living standards and economic development. These factors have played a key role in decreasing the prevalence of *Helicobacter pylori*, the main risk factor for gastric cancer. Additionally, national screening programs in high-incidence countries such as Japan and South Korea have significantly contributed to reducing mortality from this tumor [3]. Despite these improvements, there is a concerning rise in the number of young people being diagnosed with gastric cancer in high-income countries, indicating a potential change in the epidemiological patterns of the disease [2,3].

In terms of histology, adenocarcinoma is the most common form of gastric cancer. It can be further divided into three groups according to the Lauren classification: intestinal type, diffuse type, and undetermined or unclassifiable. Another classification based on tumor location distinguishes between cardia (upper stomach) and non-cardia (lower stomach) gastric cancer [3].

The etiology of gastric cancer is multifactorial, with *Helicobacter pylori* infection recognized as the main risk factor for the non-cardia form [2,3]. The disease is believed to start with chronic gastritis, which then progresses to gastric atrophy, intestinal metaplasia, and dysplasia, ultimately leading to cancer [3]. Dietary habits, such as high salt intake, consumption of processed foods, and low intake of fruits and vegetables, have been identified as risk factors. Other lifestyle behaviors like cigarette smoking and alcohol consumption are also associated with an increased risk. Additional risk factors include pernicious anemia, previous gastric surgery, family history of gastric cancer, and living in a high-incidence area [2,3,4]. In contrast, for cardia gastric cancer, overweight and gastroesophageal reflux disease have been identified as significant risk factors [1,3]. Approximately 10% of gastric cancer cases have a family history of the disease, but only 1–3% are associated with hereditary cancer syndromes. The most important one is Hereditary Diffuse Gastric Cancer (HDGC), in which 30–40% of patients have germline mutations in the *CDH1* gene, which encodes for the E-cadherin protein. Due to the high risk of developing a malignant neoplasm, prophylactic total gastrectomy is often recommended for these patients. Other syndromes associated with gastric cancer include Lynch syndrome (*MLH1*, *MLH2*, *PMS2*, and *MSH6*), juvenile polyposis syndrome (*BMPR1A* and *SMAD4*), Peutz-Jeghers syndrome (*STK11*), and familial adenomatous polyposis (*APC*), among others [3].

In addition to these biological and clinical factors, the incorporation of a sex and gender perspective has gained increasing attention in medicine in recent years. Sex refers to biological attributes, including chromosomal complement, reproductive anatomy, and hormonal profiles, which can influence carcinogenesis, tumor biology, immune function, and response to therapy. In gastric cancer, these factors may affect patterns of genetic and epigenetic alteration and the impact of endogenous hormones on tumor development and progression. In contrast, gender refers to socially constructed roles, behaviors, identities, and power relations that can shape exposures, healthcare access, and health-related decisions [5]. In this review, we use “sex” when the original data refer to male/female or to plausible biological mechanisms, and “gender-related” when discussing socially mediated behaviors or exposures such as smoking, alcohol consumption, diet, occupation, socioeconomic status, and healthcare-seeking behavior. Because most included studies did not explicitly distinguish sex from gender, we use “sex- and gender-related” when the distinction cannot be reliably inferred from the source data.

Despite being recognized as important, the systematic integration of a sex and gender perspective in medical research and clinical practice remains insufficient. Neglecting these dimensions may hinder the development of personalized approaches, which are particularly relevant in oncology. For example, males may have a higher predisposition to cancer due to a combination of genetic programming and the influence of sex hormones after puberty, as well as gender-specific behaviors that contribute to cancer risk [6]. As observed in other tumors of the gastrointestinal tract, the incidence of gastric cancer is significantly higher in men, who have approximately twice the risk of developing gastric cancer compared to women, suggesting that estrogen exposure may play a protective role [1,3,7].

The influence of sex and gender on gastric cancer prognosis, diagnosis, and treatment outcomes is still a controversial issue. This is partly due to the inconsistencies in published results and the fact that most studies have not been specifically designed to identify statistically significant differences related to sex and gender [7,8]. Additionally, women are often underrepresented in gastric cancer research [9]. As a result, important questions remain unanswered [10].

This lack of appropriate methodology limits our understanding of potential differences between men and women and highlights the need for clinical trials that disaggregate data by sex and gender. In the future, personalized approaches to cancer prevention and treatment are expected to benefit from considering these variables. Therefore, the aim of the current study was to analyze sex and gender differences among incidence rates, clinicopathological characteristics, risk factors, treatment outcomes, and overall survival in patients with gastric cancer through a systematic review of the available literature. Understanding how sex and gender influence gastric cancer is crucial for developing more personalized approaches to prevention, diagnosis, and treatment, and further studies are needed to explore the underlying mechanisms of these differences.

## 2. Methods

### 2.1. Search Strategy

To conduct this study, a broad, comprehensive, and reproducible search focused on sex- and gender-related differences in patients with gastric cancer was performed in the main medical bibliographic databases (PubMed, Embase and Web of Science). The systematic search was carried out in February 2026 following the PRISMA 2020 guidelines [11] and included all articles published in English from database inception to the search date. The completed PRISMA 2020 checklist is provided as Supplementary Tables S1 and S2. The review was not prospectively registered, and no formal review protocol was prepared. Data extraction was performed independently by two reviewers. Extracted variables included study design, country, sample size, clinicopathological characteristics, incidence data, risk factors, treatment outcomes, and survival outcomes. Discrepancies were resolved by consensus with senior investigators.

The search strategy was adapted from the methodology proposed by Song et al. [12]. The most effective combination of terms for identifying relevant studies was the following: (sex based OR sex factors OR sex distribution OR sex characteristics OR sex dimorphism OR gender difference OR gender based) AND (gender[ti] OR sex[ti] OR women[ti] OR female[ti]) AND (stomach neoplasms/diagnosis OR stomach neoplasms/epidemiology OR stomach neoplasms/etiology OR stomach neoplasms/mortality OR stomach neoplasms/prevention and control) AND (Humans[Mesh] AND English[lang]). This strategy intentionally prioritized studies in which sex or gender was a central analytical focus. We acknowledge that it may have missed studies that reported sex-stratified results only as secondary analyses without mentioning sex- or gender-related terms in the title. To reduce this potential selection bias, we manually screened the reference lists of relevant systematic reviews, meta-analyses, and included articles to identify additional eligible studies not captured by the electronic search.

Before proceeding with the selection of articles, the inclusion and exclusion criteria were clearly defined.

### 2.2. Inclusion Criteria

This systematic review included original studies involving both male and female patients diagnosed with gastric cancer. In order to be eligible, studies were required to explicitly analyze sex and/or gender differences in at least one relevant domain, such as incidence, prevalence, clinical or pathological characteristics, risk factors, prognosis, survival outcomes, treatment response, or healthcare disparity. A variety of study designs were considered, including randomized controlled trials, cohort studies, case–control studies, cross-sectional studies, and ecological studies. Only studies published in English, involving human participants, and focused on gastric adenocarcinoma were included. However, one study including esophageal cancer was retained due to the relevance of the information provided [9].

### 2.3. Exclusion Criteria

Studies were excluded if they did not consider sex or gender as a variable of analysis, or if they were studies of only one sex or gender identity. Additionally, certain publication types were excluded, such as case reports, editorials, commentaries, letters to the editor, systematic reviews, meta-analyses, and preclinical studies. Articles focusing on molecular, genetic, or immunological mechanisms were not considered either. The aim was to prioritize evidence with potential implications for clinical practice, healthcare planning, or public health strategies, taking sex and gender into account.

### 2.4. Data Synthesis

Given the marked heterogeneity in study design, populations, outcomes, and reported measures, a qualitative synthesis was performed. Studies were grouped according to the main analyzed domains, including incidence, clinicopathological characteristics, risk factors, treatment outcomes, and survival. Because the included studies were predominantly observational and heterogeneous, no meta-analysis was attempted. A formal risk-of-bias, reporting-bias, or certainty-of-evidence assessment was not performed. Instead, the interpretation was kept narrative and cautious, with attention to study design, confounding, outcome definitions, and generalizability.

## 3. Results

### 3.1. Study Selection

**Figure 1:**
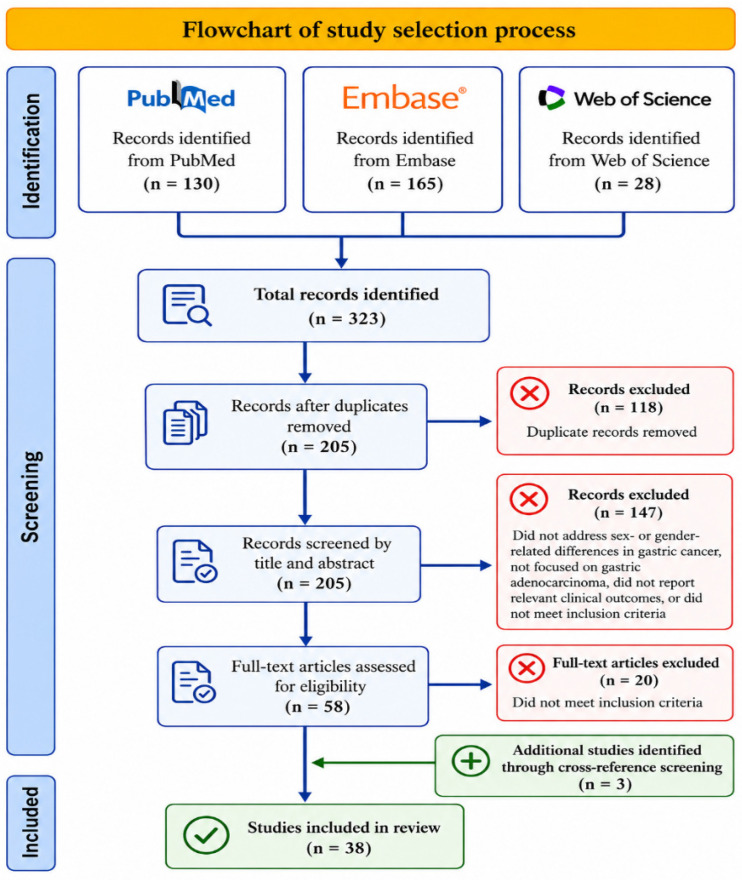
Flowchart of study selection process. The systematic search identified 323 records: 130 from PubMed, 165 from Embase, and 28 from Web of Science. After removal of 118 duplicate records, 205 articles underwent title and abstract screening. Of these, 147 were excluded because they did not specifically address sex- or gender-related differences in gastric cancer, were not focused on gastric adenocarcinoma, did not report relevant clinical outcomes, or did not meet the predefined inclusion criteria. Fifty-eight full-text articles were assessed for eligibility, resulting in the exclusion of 20 additional studies. Furthermore, 3 additional studies were identified through cross-reference screening of relevant systematic reviews and meta-analyses and were included in the final analysis. Ultimately, 38 studies met all inclusion criteria and were included in this review.

The study selection process for this systematic review is illustrated in Figure 1. The systematic search identified 323 records: 130 from PubMed, 165 from Embase, and 28 from Web of Science. After duplicate removal and title and abstract screening, potentially eligible articles were selected for full-text assessment. Most records retrieved from Embase and Web of Science were excluded because they did not specifically address sex- or gender-related differences in gastric cancer, were not focused on gastric adenocarcinoma, did not report relevant clinical outcomes, or overlapped with studies already identified through PubMed. Three additional eligible studies were identified through Embase. Reference list screening of relevant systematic reviews and meta-analyses identified 3 additional studies. Ultimately, 38 studies met all inclusion criteria and were included in the final systematic review.

The majority of studies included were observational studies, mainly retrospective in design. Table 1 provides an overview of the characteristics of each included study, as well as a summary of their main findings.

### 3.2. Sex- and Gender-Related Differences in Incidence Rates

Several studies included in this review compared the incidence of gastric cancer between men and women [2,13,17,25,36,39,41,45]. Most studies reported a general decline in incidence rates over recent decades. However, Cayuela et al. [2] observed a recent increase in incidence specifically among young men.

Overall, most included studies consistently observed a higher incidence of gastric cancer in men, although the magnitude of this difference varied by age, anatomical site, and histological subtype [2,13,18,19,24,25,33,35,36,39,41,45]. For example, Yao et al. [45] reported that the male-to-female incidence ratio was more pronounced in cardia gastric cancer (4.2:1) than in non-cardia gastric cancer (1.6:1). This sex and gender difference followed a “low–high–low” curve: similar ratios in youth, an increase after 50–60 years of age, and a decline in older age as incidence rates women become closer to those in men after menopause [36,45]. When temporal trends were analyzed, some studies described an increasing male-to-female incidence ratio, indicating a growing disparity [18,25]. In contrast, other studies reported that the incidence gap between men and women had narrowed over time, particularly for non-cardia gastric cancer, likely due to a more pronounced decline among men [41,45].

Subgroup analyses revealed that, although men generally have higher overall incidence, younger women may experience higher burden within specific age groups or tumor subtypes, particularly diffuse-type or advanced-stage disease [17,39]. Intestinal gastric cancer, which is associated with environmental factors such as *Helicobacter pylori* infection, dietary habits, and cigarette smoking, was more commonly reported in men. In contrast, diffuse gastric cancer, with less well-defined environmental risk factors and a more important hereditary component, was more frequently reported in young women in several cohorts [2,17,45]. This pattern is consistent with the findings by Corso et al. [17], who focused exclusively on diffuse-type cancer and observed higher incidence rates among women.

Finally, socioeconomic status may contribute to sex- and gender-related differences in gastric cancer incidence. According to Lou et al. [25], the disparity between men and women was more pronounced in countries with higher socioeconomic status. However, Aguilar et al. [13] found that, within Spain, only men living in the most deprived areas had an increased risk of developing gastric cancer, while this association was not observed in women.

### 3.3. Baseline Clinicopathological Characteristics Based on Sex and Gender

A detailed overview of the baseline clinicopathological characteristics disaggregated by sex/gender is provided in Table 2. Most studies reported a mean age around the sixth decade of life, although this varied widely across the included studies, ranging from 47.0 [22] to 71.8 [44] years in men and from 43.1 [47] to 72.6 years [44] in women. Regarding body mass index (BMI), most studies did not report this variable in detail; in those that did, the mean BMI was borderline between normal weight and overweight, with a mean of around 25 kg/m^2^. Plazas et al. [9], for example, reported BMI values ranging from 13 to 48 kg/m^2^ in both men and women.

In terms of tumor characteristics, this stage was most commonly reported according to the TNM classification, although this varied widely between studies. For example, some studies classified disease extent as localized, regional, or disseminated. In studies focusing on specific patient groups, all of the participants presented with the same stage, which was either advanced [9] or early-stage [15].

Tumor location was analyzed by several studies, but this variable was difficult to compare due to the use of different classification criteria [4,9,14,21,22,26,27,28,29,30,37,43,44,45]. While many studies divided tumors into upper, middle, and lower thirds of the stomach, others only distinguished between cardia and non-cardia cancer. Cardia gastric cancer refers to tumors located in the proximal (upper) part of the stomach, adjacent to the gastroesophageal junction, whereas non-cardia gastric cancer refers to tumors arising in the distal (lower) portions of the stomach [1,3]. Most studies reported a higher proportion of non-cardia subtype in both men and women. However, in one study, the proportion of cardia and non-cardia subtypes was similar, particularly among men [45], and in another study, all included tumors were located at the gastroesophageal junction according to the study design [29]. Across the studies that reported the anatomical site, no consistent sex/gender-associated pattern in tumor location was evident.

Finally, histological subtype was also considered in several studies, with tumors mainly classified according to the Lauren classification [4,9,21,23,26,28,34,38,47] or in terms of differentiation [14,20,22,27,29,30,43,44]. The analysis revealed a consistent pattern in which the intestinal type was more frequent in men, ranging from 35.9% [28] to 69.6% [4]. Similar findings were reported by Schildberg et al. [35], who observed that male patients more frequently presented with intestinal-type tumors, whereas women more often presented with diffuse and poorly differentiated histology. In contrast, the diffuse type was more frequently reported in women, ranging from 9.5% [28] to 56.9% [38]. Yu et al. [47] and Corso et al. [17] conducted their studies focusing exclusively on diffuse-type and reported higher incidence rates in women. Moreover, in one of the largest surgical cohorts included in this review, Choi et al. [4] reported diffuse histology in approximately half of female patients compared with one quarter of male patients. Tumors in women also tended to be more poorly differentiated [14,20,22,27,30,43], whereas well-differentiated tumors were more frequently observed in men [14,20,22,27,29,30,44].

### 3.4. Risk Factors for Gastric Cancer Based on Sex and Gender

**Figure 2:**
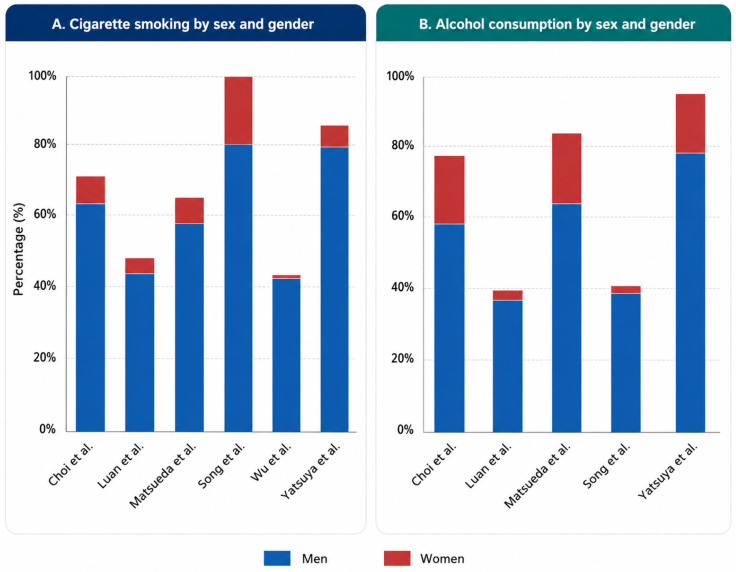
Prevalence of lifestyle-related risk factors according to sex and gender across the included studies. (**A**) Percentages represent the proportion of patients with a current or previous history of cigarette smoking reported in each study. (**B**) Percentages represent the proportion of patients with a current or previous history of alcohol consumption reported in each study [16,26,29,37,43,46].

Several studies analyzed risk factors associated with gastric cancer [4,13,18,20,25,26,29,30,31,37,41,43,46,47]. Cigarette smoking was the most frequently reported risk factor (Figure 2A), with prevalence rates of current or former smokers ranging from 44% [26] to 81.3% [37] among men, while the highest reported percentage among women was 27% [37]. Alcohol consumption showed a similar pattern (Figure 2B), with the proportion of current or former drinkers ranging from 34.9% [26] to 78.1% [46] among men, while the highest reported rate among women was 20.2% [37]. These variables are best interpreted as gender-related exposures, although they may also interact with biological sex through metabolic, hormonal, and inflammatory pathways.

Other notable risk factors included *Helicobacter pylori* infection and family history of gastric cancer, which was defined as having at least one first-degree relative affected [46]. Choi et al. [4] and Luan et al. [26] reported similarly high prevalence rates of *H. pylori* infection between sexes (53.7–55.7% in men vs. 51.6–61.2% in women). However, family history showed more variability. Choi et al. [4] found a higher prevalence in men (17%) compared to women (7.8%), while Yatsuya et al. [46] reported similar rates (9.5% in men vs. 10.5% in women).

In addition to these well-known risk factors, some studies highlighted the role of socioeconomic status in gastric cancer incidence and outcomes [13,18,25,41,42]. Most of these studies observed higher incidence and mortality rates in patients living in regions with lower socioeconomic status, but sex- or gender-specific patterns were not consistently reported. In contrast, Lou et al. [25] found that the disparity between men and women was more pronounced in developed countries.

### 3.5. Treatment Selection and Postoperative Complications

Several studies identified sex-associated differences in treatment approaches and postoperative outcomes of gastric cancer [9,14,21,22,32]. While overall surgical rates were similar between men and women, some specific differences were reported. For example, Kalff et al. [21] showed that women were more likely to undergo partial gastrectomy (55.8% vs. 48.8%) and less likely to receive perioperative treatment (35.2% vs. 40.3%), although these differences were not significant after adjustment for clinicopathological variables. According to Kim et al. [22], surgical methods, including gastric resection, gastrojejunal bypass, and no surgery, were significantly different between younger and older women, but not between age groups in men.

In terms of complications, male patients were found to have a higher rate of postoperative complications in several studies [14,21,26,38,43]. Furthermore, men had more high-risk comorbidities (71% vs. 61%) [14] and more re-interventions (16.2% vs. 11.9%) [21], leading altogether to a poorer prognosis. However, this pattern was not uniform. Sah et al. [32] reported worse postoperative outcomes in female patients, including longer hospital stays and more severe complications. These findings should therefore be interpreted cautiously, as postoperative outcomes may be influenced by baseline comorbidity, surgical extent, institutional practices, and case-mix.

### 3.6. Overall Survival

Although some studies did not find significant sex- or gender-related differences in overall survival [9,29], many reported outcome differences, although the direction and magnitude varied. Some studies found that female patients had significantly better overall survival, particularly in early-stage disease [4,14,44]. Similarly, Li et al. [24]. analyzed 99,922 gastric cancer cases from the SEER database and found that women generally had superior cancer-specific survival compared with men, although these differences varied according to age and tumor stage. These poorer outcomes in men were associated with a higher comorbidity burden [14,21,43] and lower visceral-to-subcutaneous fat ratio [20].

Conversely, some analyses suggested equal or more favorable outcomes in men within specific subgroups. For example, Kalff et al. [21] reported inferior 5-year relative survival in women compared with men (48.6% vs. 55.8%), despite lower postoperative complication rates. Similarly, Sun et al. [40] found that younger women had similar or even higher mortality rates compared with men. The survival advantage for women generally diminished in advanced stages or after adjustment for tumor characteristics [4,34]. Additionally, some studies described a significant interaction between sex and age, observing poorer outcomes in young women [4,22,23,27]. Supporting these observations, Choi et al. [16], in a large-scale analysis including 14,739 patients, showed that survival differences according to sex were strongly modified by age, with younger women presenting less favorable outcomes despite the overall survival advantage usually observed in females. Histological subtype was another important factor to consider, as women with signet-ring cell carcinoma had significantly worse survival [23]. The obesity paradox, in which overweight or obese patients sometimes had better survival rates than those with normal or low body weight, was observed in men but not in women [26]. Overall survival should be interpreted with caution because it can be influenced by age, comorbidities, stage at diagnosis, treatment access, and non-cancer-related mortality.

Finally, temporal trends in gastric cancer mortality according to sex and gender were also analyzed. García-Esquinas et al. [18] observed a steeper decline in mortality among women compared to men (−3.65% vs. −2.90%), a pattern that was also confirmed by Song et al. [39], who reported an even greater annual reduction in mortality for both sexes (−5.9% vs. −4.3%).

## 4. Discussion

This systematic review analyzed 38 studies and more than 500,000 patients with gastric cancer to synthesize sex- and gender-related differences across multiple aspects of the disease. The review provides a comprehensive synthesis of prognosis and complications together with incidence, risk factors, and sociocultural determinants, offering a broader perspective on sex- and gender-related differences in gastric cancer that has not been addressed in this way before. Because the included evidence was heterogeneous and largely observational, the findings should be interpreted as a qualitative and hypothesis-generating synthesis rather than as definitive causal evidence.

In terms of incidence, this review suggests that gastric cancer is more commonly reported in men than in women. Although further research is needed to fully understand the underlying biological mechanisms, existing hypotheses suggest a protective role of estrogens in premenopausal women that could explain the 10–15 year delay in the onset of intestinal-type gastric cancer [36]. Several meta-analyses support this hypothesis, indicating that estrogen exposure, both endogenous and exogenous, is associated with a lower risk of gastric cancer [48,49]. This protective effect may be partly mediated by modulation of the inflammatory response induced by *H. pylori*, reducing the production of proinflammatory cytokines (IFN-γ, TNF-α, IL-1β) and increasing anti-inflammatory mediators such as IL-10, thereby influencing the inflammatory microenvironment, which is crucial to gastric carcinogenesis [48]. Additionally, the expression of the estrogen receptor ERβ in gastric tissue has been associated with less aggressive tumor phenotypes (intestinal type, lower tumor stage, and less perineural invasion) and better survival outcomes, while its absence predicts a poorer prognosis. The activation of the ERβ and GPER receptors appears to have protective effects, whereas the role of ERα remains more controversial [49].

**Figure 3:**
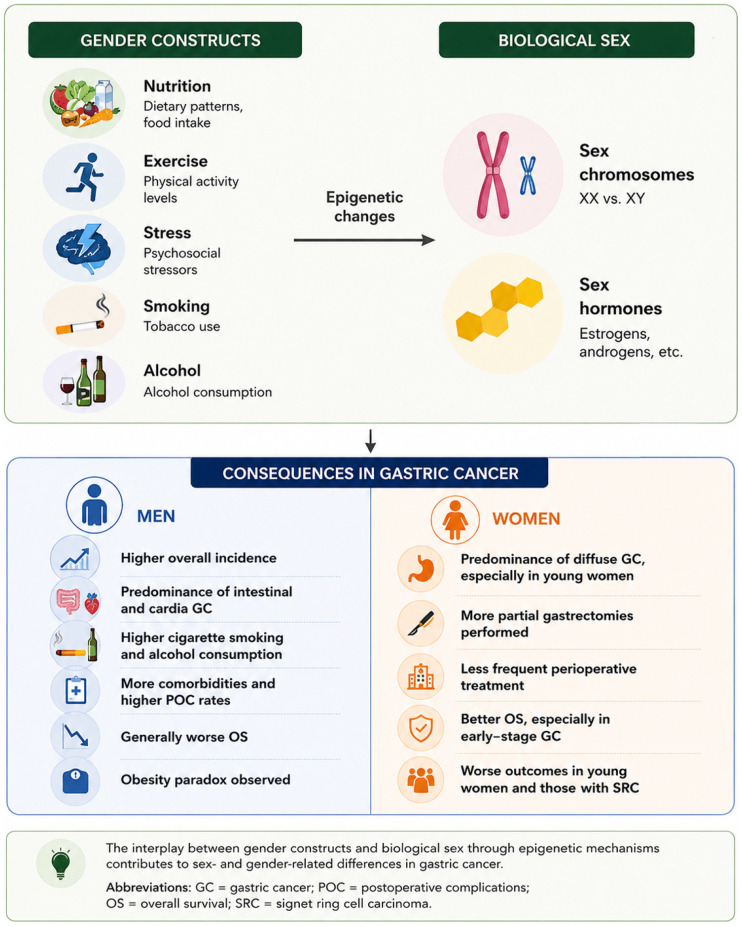
Interaction between biological sex and gender-related factors and their influence on gastric cancer characteristics and outcomes. The figure summarizes how biological determinants (sex chromosomes and sex hormones) and gender-associated factors, including lifestyle habits and environmental exposures, may contribute to sex- and gender-related differences in gastric cancer incidence, histological subtype distribution, treatment patterns, postoperative complications, and survival outcomes. Men show a higher overall incidence of gastric cancer, greater exposure to smoking and alcohol consumption, and generally poorer overall survival in several cohorts, whereas women more frequently present with diffuse-type and signet-ring cell gastric cancer, particularly at younger ages. These patterns should be interpreted as conceptual associations rather than causal relationships. The upper conceptual framework was adapted from Mauvais-Jarvis et al. [6]. Abbreviations: GC = gastric cancer; POC = postoperative complications; OS = overall survival; SRC = signet-ring cell carcinoma.

We observed marked differences in the prevalence of key gastric cancer risk factors, particularly smoking and alcohol consumption, which were consistently higher among men. These findings align with prior research indicating that gender-related behaviors, shaped by cultural norms and socioeconomic context, contribute substantially to the gastric cancer risk profile. Gender-related factors can influence health perceptions, healthcare-seeking behavior, and interactions with medical professionals [6]. Moreover, behaviors and exposures, such as dietary habits, stress management, smoking, and physical activity, may interact with biological sex and induce epigenetic modifications, thereby influencing disease risk and progression (Figure 3). In several studies, obesity was also more strongly associated with higher socioeconomic status in men, potentially explaining the faster BMI growth rates observed in male populations in high-income settings. Such patterns may be compounded by differential access to healthcare and screening, which can amplify disparities in early detection. Considering all these differences, targeted screening of high-risk populations rather than mass population screening may be more cost-effective in many countries [25].

The predominance of diffuse-type and poorly differentiated tumors in younger women also warrants a mechanistic interpretation. Diffuse and signet-ring cell gastric cancers are characterized by impaired epithelial cohesion and altered differentiation programs, with involvement of adhesion-related pathways such as CDH1/E-cadherin and beta-catenin, as well as molecular alterations including RHOA pathway changes and CLDN18-ARHGAP fusions [50]. Recent immunohistochemical studies further suggest that EMT-related phenotypes and adhesion/differentiation markers may help contextualize the aggressive clinicopathological behavior of diffuse gastric cancer. In particular, loss or altered localization of VSIG1, E-cadherin, and beta-catenin has been associated with advanced stage and a high lymph-node ratio, while mucin-phenotype markers such as MUC2, MUC5AC, and CDX2 may reflect gastric versus intestinal differentiation programs [51,52]. These observations are biologically plausible but should not be interpreted as proof of sex-specific causality, because most molecular studies have not been designed to evaluate sex-stratified mechanisms.

While differences in incidence are relatively well established, the impact of sex and gender on gastric cancer prognosis remains more controversial. Although the meta-analysis conducted by Luan et al. [7] reported better 3-year and 5-year survival for women, the evidence is inconsistent across studies analyzed in our review. We observed that women often had better survival in unselected or early-stage cohorts, whereas younger women and those with diffuse or signet-ring cell histology sometimes experienced worse outcomes. This could be partly explained by the higher prevalence of the diffuse subtype in young women, as it is known to be more aggressive and difficult to diagnose and has a poorer response to conventional treatments [50]. However, survival differences may also reflect confounding by age, comorbidities, stage at diagnosis, treatment access, non-cancer-related mortality, and molecular subtype. Therefore, sex- and gender-related survival patterns should be interpreted cautiously.

Evidence regarding sex- and gender-related differences in treatment response also remains limited. Some studies have shown higher postoperative complication rates in men, possibly due to a greater burden of preoperative comorbidities and more extensive surgeries [21]. Biological sex may also influence the pharmacokinetics of chemotherapy drugs through differences in body composition, drug-metabolizing enzyme expression, and drug–erythrocyte binding, potentially resulting in higher dose intensities in women [10]. However, the persistent underrepresentation of women in gastric cancer research limits our understanding of sex- and gender-specific treatment outcomes, reflecting a broader issue in biomedical research [9].

This significant historical bias has contributed to the limited applicability of evidence to female patients. Much of what healthcare professionals know about disease diagnosis, treatment, and prevention is based on studies conducted primarily in male cells, male mice, and men [53]. This bias is rooted in the mistaken belief that females introduce greater experimental variability due to cyclic hormonal fluctuations and from the historical assumption that no meaningful sex differences exist beyond reproductive function [5]. Incorporating sex and gender analysis into research can improve experimental reproducibility and efficiency, help to reduce bias, promote social equity in scientific outcomes, and drive opportunities for discovery and innovation [8]. In this context, the are some institutions [5] that have developed tools to guide researchers in incorporating a sex and gender perspective throughout all phases of biomedical and health research, from problem identification to dissemination of results; for example, the PROGENERES Decalogue, which is a set of recommendations designed to assist researchers across a wide range of settings, from preclinical to clinical research and from observational studies to clinical trials [54]. Building on these recent methodological advances, we aimed to address key gaps identified in the existing literature. Our findings were partially consistent with the only previous systematic review and meta-analysis on the topic, which was conducted by Luan et al. [7]. That study focused on clinicopathological characteristics, prognosis, postoperative complications, and metastasis but did not thoroughly examine risk factors, an aspect that appears to be of crucial importance when explaining the differences between men and women. Furthermore, the authors used the terms “sex” and “gender” interchangeably, which is understandable given the data limitations, but it is important to distinguish between these concepts whenever possible.

Our review has several strengths. To our knowledge, it is one of the most comprehensive syntheses to date of sex- and gender-related differences in gastric cancer, encompassing incidence, clinicopathological features, risk factors, treatment patterns, and survival. By distinguishing, as much as possible, between biological sex-related mechanisms and gender-related exposures, it offers a multidimensional framework that is less frequently addressed in this disease. The inclusion of studies from different time periods and geographic regions enables the identification of both consistent trends and context-specific patterns. Furthermore, the structured synthesis and detailed summary tables facilitate appraisal of the available evidence and may serve as a useful resource for clinicians and researchers. While important knowledge gaps remain, we hope that our work will help guide future studies toward a more systematic integration of sex and gender variables in gastric cancer research.

However, the inclusive approach of our review also introduced several limitations. Although the literature search, study selection and data extraction process were independently reviewed by two investigators and subsequently supervised by two senior investigators, the review was not prospectively registered, and no formal protocol was published. In addition, no formal risk-of-bias, reporting-bias, or certainty-of-evidence assessment was performed. This limits the reliability and certainty of the conclusions and should be considered when interpreting the findings. Moreover, there was considerable heterogeneity among the included studies in terms of inclusion criteria, study design, populations analyzed, statistical methods, and outcome definitions, which limited the possibility of conducting a quantitative synthesis through meta-analysis. Most studies were retrospective and observational, increasing the risk of selection bias, information bias, and residual confounding. Stratification by sex and gender was not consistently applied, which weakens the strength of comparisons. In this review, the terms man/woman and male/female were sometimes used according to the terminology of the original studies, reflecting the lack of differentiation between biological sex and sociocultural gender in most source articles. Additionally, many studies were conducted in Asian populations, limiting the generalizability of the findings to other geographic and ethnic groups. Finally, definitions of key variables were not always standardized, and some studies lacked sufficient detail for subgroup analyses. The subgroup analyses reported across the included studies should therefore be considered exploratory and hypothesis-generating, and they are insufficient to inform clinical or regulatory decision-making without better designed comparative studies.

## 5. Conclusions

In conclusion, this systematic review highlights relevant sex- and gender-related differences in gastric cancer incidence, clinicopathological characteristics, risk factors, treatment outcomes, and survival. Men showed higher overall incidence rates in most studies, whereas young women were more frequently affected by diffuse-type and poorly differentiated tumors. Gender-related behaviors and socioeconomic factors appear to influence risk, while biological mechanisms, including hormonal pathways, adhesion-related alterations, epithelial–mesenchymal transition, and differentiation programs, may contribute to observed clinicopathological differences. However, data on treatment and survival remain heterogeneous and limited by retrospective study designs, insufficient sex/gender disaggregation, and lack of standardized outcome reporting. Integrating a sex and gender perspective across all research phases is therefore essential for the development of more precise and equitable gastric cancer prevention and treatment strategies.

## Figures and Tables

**Table 1:** Characteristics of the studies included in the systematic review.

Author	Year	Country	Sample	Study Design	Analyzed Aspects	Summary of Main Results
Aguilar et al. [13]	2013	Spain	-	Ecological study	Incidence, risk factors	From 1993 to 2002, men living in the most deprived areas had twice the risk of GC, while SES had no effect on women. In contrast, rurality was linked to higher GC risk only in women, particularly in the most rural areas.
Arakawa et al. [14]	2023	Japan	295	Retrospective cohort study	Survival	In elderly patients, males had more upper gastric tumors, more POC, and worse OS than females. Male sex was independently associated with poorer prognosis, indicating that limited surgery may benefit high-risk elderly men.
Bando et al. [15]	2004	Japan	4231	Retrospective cohort study	Survival	In early GC, cancer-specific survival remained high, but OS declined with age, especially in patients over age 80. Age was the strongest prognostic factor, with non-cancer-related mortality increasing with age, while tumor recurrence rates remained unaffected.
Cayuela et al. [2]	2025	Spain	-	Ecological study	Incidence	From 1990 to 2019, GC cases in Spain rose slightly, but ASIR declined by 1.8% annually for both sexes, with men maintaining a higher burden than women. Incidence recently increased in young men (age 25–34), suggesting emerging sex-related differences.
Choi et al. [4]	2022	South Korea	2983	Retrospective cohort study	Risk factors, survival	Females were younger and had more diffuse-type tumors, while males had more intestinal-type and non-GC-related deaths. Females had better OS, but cancer-specific survival was similar, except in advanced stages, where females showed worse outcomes.
Choi et al. [16]	2025	South Korea	14,739	Retrospective cohort study	Survival	Survival differences according to sex were influenced by age, with younger women showing less favorable outcomes despite the overall female survival advantage.
Corso et al. [17]	2024	Italy	1094	Retrospective observational study	Incidence	From 1995 to 2021, diffuse GC made up 10.2% of cases, and was more common in individuals under age 45, particularly in women (34.0% vs. 25.7%). While overall incidence declined in the last decade, the percentage of diffuse GC increased, especially among young women.
García-E. et al. [18]	2009	Spain	-	Ecological study	Risk factors, survival	From 1976 to 2005, GC mortality declined more in women than in men (3.65% vs. 2.90%). This sex difference was especially evident in certain inland and northern regions of Spain, and a change in the rate of decline was observed for both sexes in the early 1980s.
Griffith et al. [19]	1968	UK	-	Ecological study	Survival	From 1958 to 1963, GC mortality showed a consistent “low–high–low” male-to-female ratio across ages, being approximately equal in young adults, peaking around ages 50–59, and then declining in older age. This pattern is likely to be explained by incidence data.
Hashimoto et al. [20]	2024	Japan	459	Prospective cohort study	Risk factors, survival	In males, a low VSR was associated with significantly worse OS and RFS and was an independent predictor of poor prognosis. Among females, a high VSR was linked to significantly lower RFS and predicted worse outcomes, with no difference observed in OS.
Kalff et al. [21]	2022	Netherlands	2072	Retrospective cohort study	Treatment, survival	Females undergoing surgery for GC had lower postoperative morbidity and fewer re-interventions compared to males. However, despite these advantages, females had significantly worse 5-year relative OS.
Kim et al. [22]	2008	Korea	1299	Retrospective cohort study	Treatment, survival	In males, younger patients had more undifferentiated tumors but better 10-year OS, while in females, tumor differentiation and surgical approaches varied by age, with older women showing slightly better OS. Tumor stage and sex were independent predictors of OS.
Kim et al. [23]	2016	South Korea	4722	Retrospective cohort study	Survival	Females were younger and had more poorly differentiated adenocarcinoma and SRC. They also had poorer OS, particularly in advanced cases under age 45, with SRC worsening outcomes and showing distinct ER-β expression patterns by sex.
Li et al. [24]	2020	USA	99.922	Population-based retrospective cohort study	Survival	Female patients showed better cancer-specific survival overall, although survival differences varied according to age and tumor stage.
Lou et al. [25]	2020	Global	-	Ecological study	Incidence, risk factors	From 1990 to 2017, global GC incidence declined in both sexes, but the male-to-female ratio of ASIR increased from 1.86 to 2.20, with the largest relative difference observed in the 65–69 age group. Sex differences in ASIRs were associated with higher HDI levels.
Luan et al. [26]	2024	China	29,779	Prospective cohort study	Risk factors, survival	Females showed better OS than males, though smoking history worsened prognosis among women. In men, heavy tobacco use significantly worsened OS, and an obesity paradox was noted, with a higher BMI associated with better survival outcomes.
Maehara et al. [27]	1992	Japan	1031	Retrospective cohort study	Survival	Women under age 50 had lower 10-year OS than men, with key prognostic factors (operative curability, lymph node metastasis, depth of invasion, and tumor size) differing between sexes. Early detection and postoperative CTX may benefit those with advanced disease.
Maguire et al. [28]	1996	Spain	851	Retrospective cohort study	Survival	Women showed better OS after adjusting for tumor stage and age, especially in Barcelona at the local stage, but this survival advantage varied by location (Mataró and Soria), suggesting the sex-based survival benefit may be influenced by tumor stage.
Matsueda et al. [29]	2024	Japan	188	Mixed cohort study	Incidence, risk factors	The incidence of adenocarcinoma of the GEJ remained stable among female and young adult patients. These groups of patients without associated risk factors had higher rates of undifferentiated tumors compared to patients with risk factors or older age groups.
Nam et al. [30]	2021	South Korea	5961	Retrospective cohort study	Risk factors, survival	Overall mortality was lower in women, especially over age 60, with a BMI below 25 kg/m^2^ and at stage I disease. In men, mortality rose with age and a low BMI, while in women, it was higher at extreme ages (<40 and ≥70 years) but lower with a BMI ≥30 kg/m^2^.
Nam et al. [31]	2022	South Korea	29,775	Prospective cohort study	Risk factors, survival	A low BMI significantly increased the risk of GC and all-cause mortality, with risk thresholds at 23 kg/m^2^ in men and 18.5 kg/m^2^ in women. High fasting glucose was associated with increased gastric cancer risk only in women.
Plazas et al. [9]	2022	Spain	2993 *	Retrospective cohort study	Treatment, survival	Women with advanced disease had tumors more often HER2-negative, grade 3, diffuse, and SRC histology, with peritoneal rather than liver spread, and higher toxicity during CTX. Despite these differences, there were no significant sex-based differences in PFS or OS.
Sah et al. [32]	2009	China	357	Retrospective cohort study	Treatment	Females experienced higher rates and severity of POC, as well as longer hospital stays, after GC surgery. Gender was identified as an independent risk factor for early POC, highlighting its influence on surgical outcomes.
Sandler et al. [33]	1987	USA	-	Ecological study	Survival	From 1950 to 1979, age-adjusted sex-specific mortality rates for GC in white Americans showed an increasing male-to-female ratio, rising from 1.8 to 2.1, despite a decline in crude ratios. The overall decline in GC mortality affected both sexes equally.
Sato et al. [34]	2009	Japan	72,789	Retrospective cohort study	Survival	Women showed worse OS, but after adjusting for disease stage, histological type, detection method, and treatment, women actually had slightly better OS. The observed lower OS in women was mainly due to their cancers being diagnosed at more advanced stages.
Schildberg et al. [35]	2025	Germany	-	Retrospective observational study	Incidence, clinicopathological characteristics	Male patients showed higher GC incidence and more intestinal-type tumors, whereas women more frequently presented diffuse and poorly differentiated histology, particularly at younger ages.
Sipponen et al. [36]	2002	Finland	-	Ecological study	Incidence	From 1955 to 1990, GC incidence followed a “low–high–low” pattern, peaking around age 60 due to a delayed onset of intestinal-type GC in females. This consistent global pattern has remained stable over decades despite declining overall GC incidence.
Song et al. [37]	2008	Korea	13,396	Prospective cohort study	Risk factors	Alcohol consumption increased GC risk in women, particularly among former heavy drinkers. No significant association was observed in men, suggesting a sex-specific relationship between alcohol and GC that may persist in women even after ceasing consumption.
Song et al. [38]	2025	China	287	Retrospective cohort study	Survival	Males had more muscle and less subcutaneous fat, with different RFS predictors by sex: visceral fat, lymphocyte count, and T stage were key in men, while subcutaneous fat loss, albumin, and CEA levels were important in women (muscle mass and POC in both).
Song et al. [39]	2015	South Korea	-	Ecological study	Incidence	From 1999 to 2010, overall age-standardized GC incidence declined slightly in both males and females, while mortality rates dropped significantly. However, age-specific analysis revealed a concerning flat or rising incidence trend among females aged 40–54.
Sun et al. [40]	2002	Japan	-	Ecological study	Survival	From 1957 to 1997, eliminating GC deaths increased life expectancy more in men, indicating their higher overall mortality rates. However, young women exhibited a higher risk of GC death, with a stable or slightly decreasing sex ratio over four decades.
Sun et al. [41]	2018	China	87,242	Retrospective observational study	Incidence, risk factors, survival	From 1984 to 2013, females initially had higher 12-month OS, but this advantage reversed later. Cox regression analysis confirmed this shift, showing that females had a better prognosis in the first decade, but males had better survival in the following two decades.
Suryawala et al. [42]	2015	USA	285	Retrospective cohort study	Incidence	Men and women within each ethnic group had similar annual rates of GC diagnosis, contrasting with national trends where females typically had lower risks than males. African American males had higher healthcare utilization than African American females and white patients.
Wu et al. [43]	2023	China	376	Retrospective cohort study	Risk factors, survival	Comorbidities assessed by the ACCI significantly predicted lower OS in GC patients, whereas the standard CCI did not. This association between high-risk ACCI scores and increased mortality was observed in male patients but not in females.
Xing et al. [44]	2024	Japan	18,436	Retrospective cohort study	Survival	Males had more differentiated tumors and were often diagnosed through medical checkups, while females showed better OS, particularly in early-stage disease, with multivariate analysis confirming lower mortality risk.
Yao et al. [45]	2020	USA	57,534	Ecological study	Incidence	From 1992 to 2014, sex differences varied by cancer subtype, with the male-to-female ratio for cardia GC peaking at ages 55–69 and that for non-cardia GC stabilizing after age 60. Differences in non-cardia GC decreased in most races, but cardia GC remained more stable.
Yatsuya et al. [46]	2002	Japan	110,573	Prospective cohort study	Risk factors	A positive family history of GC increased the risk of death from the disease in both men and women, especially among individuals aged 40–59. Women showed a particularly high risk when multiple family members were affected or when their mother or sister had GC.
Yu et al. [47]	2011	China	192	Cross-sectional study	Risk factors	In HDGC, females were more common and diagnosed younger, with a lower male-to-female ratio than in general GC. There were generational and regional differences: parents were older than offspring at diagnosis, and Asian patients were older and more often male.

* Sample size shown for Plazas et al. [9] corresponds exclusively to gastric cancer patients. The total study population included 3274 participants. Abbreviations: GC = gastric cancer; SES = socioeconomic status; POC = postoperative complications; OS = overall survival; ASIR = age-standardized incidence rates; VSR = visceral-to-subcutaneous fat rate; RFS = relapse-free survival; SRC = signet ring cell carcinoma; HDI = human development index; BMI = body mass index; CTX = chemotherapy; GEJ = gastroesophageal junction; PFS = progression-free survival; CEA = carcinoembryonic antigen; ACCI = age-adjusted Charlson Comorbidity Index (CCI); HDGC = hereditary diffuse gastric cancer.

**Table 2:** Patient and tumor characteristics in the analyzed studies.

Study	Sex	Sample	Age (Years)	BMI (kg/m^2^)	Stage	Tumor Location	Histological Type
Arakawa et al. [14]	Men	181 (61.4%)	<85: 160 (88%) ≥85: 21 (12%)	<25: 146 (81%) ≥25: 35 (19%)	-	Upper: 61 (34%) Middle and lower: 120 (66%)	Differentiated: 125 (69%) Undifferentiated: 56 (31%)
	Women	114 (38.6%)	<85: 101 (89%) ≥85: 13 (11%)	<25: 90 (79%) ≥25: 24 (21%)	-	Upper: 19 (17%) Middle and lower: 95 (83%)	Differentiated: 59 (52%) Undifferentiated: 55 (48%)
Bando et al. [15]	Men	2852 (67.4%)	≤70: 2186 71–80: 596 >80: 70	-	100% early stage	-	-
	Women	1379 (32.6%)	≤75: 1224 76–80: 110 >80: 45	-	100% early stage	-	-
Choi et al. [4]	Men	2005 (67.2%)	Mean: 61.66 ± 11.63	-	I: 1569 (78.3%) II: 254 (12.7%) III: 143 (7.1%) IV: 39 (1.9%)	Upper: 58 (2.9%) Middle: 835 (41.6%) Lower: 1112 (55.5%)	Intestinal: 1396 (69.6%) Diffuse: 520 (25.9%) Mixed: 89 (4.5%)
	Women	978 (32.8%)	Mean: 59.36 ± 13.47	-	I: 743 (76%) II: 151 (15.4%) III: 69 (7.1%) IV: 15 (1.5%)	Upper: 19 (2%) Middle: 497 (50.8%) Lower: 462 (47.2%)	Intestinal: 447 (45.7%) Diffuse: 494 (50.5%) Mixed: 37 (3.8%)
Hashimoto et al. [20]	Men	300 (65.4%)	<65: 88 ≥65: 212	<18.5: 12 18.5–24.9: 211 ≥25: 77	I: 207 II/III: 93	-	Well/moderately differentiated: 171 Poorly differentiated: 129
	Women	159 (34.6%)	<65: 52 ≥65: 107	<18.5: 35 18.5–24.9: 100 ≥25: 24	I: 109 II/III: 50	-	Well/moderately differentiated: 55 Poorly differentiated: 104
Kalff et al. [21]	Men	1304 (62.9%)	Mean: 68.6 <55: 158 (12.1%)	Mean: 25.3	-	Fundus: 136 (10.9%) Corpus: 389 (31.2%) Antrum: 468 (37.5%) Pylorus: 104 (8.3%) Entire stomach: 73 (5.8%) Gastric remnant: 78 (6.3%)	Intestinal: 515 (57.7%) Diffuse: 311 (34.9%) Mixed: 66 (7.4%)
	Women	768 (37.1%)	Mean: 67.7 <55: 146 (19.0%)	Mean: 25.1	-	Fundus: 37 (5.0%) Corpus: 232 (31.7%) Antrum: 342 (46.7%) Pylorus: 64 (8.7%) Entire stomach: 53 (7.2%) Gastric remnant: 5 (0.7%)	Intestinal: 239 (45.0%) Diffuse: 258 (48.6%) Mixed: 34 (6.4%)
Kim et al. * [22]	Men	865 (66.6%)	Mean: 47 ± 6.4	-	I: 321 II: 104 III: 230 IV: 210	Upper third: 100 Middle third: 265 Lower third: 489 Entire: 11	Well differentiated: 495 Undifferentiated: 370
	Women	434 (33.4%)	Mean: 46.5 ± 7.2	-	I: 197 II: 61 III: 123 IV: 53	Upper third: 39 Middle third: 166 Lower third: 218 Entire: 11	Well differentiated: 155 Undifferentiated: 279
Kim et al. [23]	Men	3136 (66.4%)	Mean: 57.9 ± 11.2 ≤45: 431 (13.7%) >45: 2705 (86.3%)	-	I: 1858 (59.2%) II: 119 (3.8%) III: 1159 (37%)	-	Intestinal: 758 (59.5%) Diffuse: 416 (32.6%) Mixed: 101 (7.9%)
	Women	1586 (33.6%)	Mean: 55 ± 13 ≤45: 382 (24.1%) >45: 1204 (75.9%)	-	I: 897 (56.6%) II: 65 (4.1%) III: 623 (39.3%)	-	Intestinal: 245 (39.9%) Diffuse: 295 (48%) Mixed: 74 (12.1%)
Luan et al. [26]	Men	22,120 (74.3%)	18–34: 410 (1.9%) 35–50: 3446 (15.6%) 51–64: 11,000 (49.7%) ≥65: 7264 (32.8%)	<18.5: 1532 (7.7%) 18.5–22.9: 8400 (42.1%) 23–27.4: 7893 (39.6%) ≥27.5: 2123(10.6%)	0: 126 (0.7%) I: 3573 (20.9%) II: 3636 (21.2%) III: 7757 (45.3%) IV: 2036 (11.9%)	Proximal: 7516 (36.4%) Distal: 11,923 (57.7%) Total: 1224 (5.9%)	Intestinal: 3572 (21.5%) Diffuse: 2710 (16.3%) Mixed: 2403 (14.5%) Unknown: 7912 (47.7%)
	Women	7659 (25.7%)	18–34: 474 (6.2%) 35–50: 1836 (24.0%) 51–64: 3259 (42.6%) ≥65: 2090 (27.3%)	<18.5: 787 (11.4%) 18.5–22.9: 3182 (46.3%) 23–27.4: 2286 (33.2%) ≥27.5: 624 (9.1%)	0: 37 (0.6%) I: 1293 (21.6%) II: 1210 (20.2%) III: 2591 (43.2%) IV: 863 (14.4%)	Proximal: 1490 (20.9%) Distal: 5232 (73.5%) Total: 398 (5.6%)	Intestinal: 755 (13.1%) Diffuse: 1570 (27.2%) Mixed: 656 (11.4%) Unknown: 2792 (48.4%)
Maehara et al. [27]	Men	689 (66.8%)	Mean: 58.9 ± 11.4 ≤50: 140 (20.3%) 51–70: 450 (65.3%) ≥71: 99 (14.4%)	-	-	Upper: 207 (30%) Middle: 176 (25.5%) Lower: 306 (44.5%)	Differentiated: 347 (50.4%) Undifferentiated: 342 (49.6%)
	Women	342 (33.2%)	Mean: 55.5 ± 13.9 ≤50: 122 (35.7%) 51–70: 175 (51.1%) ≥71: 45 (13.2%)	-	-	Upper: 86 (25.1%) Middle: 118 (34.5%) Lower: 138 (40.4%)	Differentiated: 110 (32.2%) Undifferentiated: 232 (67.8%)
Maguire et al. * [28]	Men	547 (64.3%)	Mean: 66.4	-	Local: 28.9% Regional: 32.4% Disseminated: 28.7% Missing: 9.9%	Cardia: 8.1% Non-cardia: 91.9%	Diffuse: 10.2% Intestinal: 35.9% Others: 53.6%
	Women	304 (35.7%)	Mean: 69.9	-	Local: 21.9% Regional: 38% Disseminated: 29.7% Missing: 10.4%	Cardia: 4.5% Non-cardia: 95.5%	Diffuse: 9.5% Intestinal: 31.8% Others: 58%
Matsueda et al. [29]	Men	152 (80.9%)	Mean: 68 ± 12.2	<25: 101 (66%) ≥25: 51 (34%)	-	100% GEJ	Differentiated: 107 (70%) Undifferentiated: 45 (30%)
	Women	36 (19.1%)	Mean: 69.6 ± 14.0	<25: 28 (78%) ≥25: 51 (34%)	-	100% GEJ	Differentiated: 20 (56%) Undifferentiated: 16 (44%)
Nam et al. [30]	Men	3969 (66.6%)	Mean: 61.3 <40: 117 (3.0%) 40–49: 445 (12.1%) 50–59: 1067 (26.9%) 60–69: 1344 (33.9%) ≥70: 996 (25.1%)	Mean: 23.3 <18.5: 220 (5.6%) 18.5–22.9: 1569 (40.2%) 23–24.9: 1038 (26.6%) 25–29.9: 1013 (26.0%) ≥30: 62 (1.6%)	I: 2828 (71.5%) II: 357 (9.0%) III: 300 (7.6%) IV: 468 (11.8%)	Upper: 622 (15.7%) Middle: 1600 (40.38%) Distal: 1740 (43.9%)	Differentiated: 2239 (56.6%) Undifferentiated: 1717 (43.4%)
	Women	1992 (33.4%)	Mean: 59.6 <40: 159 (8.0%) 40–49: 313 (15.7%) 50–59: 430 (21.6%) 60–69: 529 (26.6%) ≥70: 561 (28.1%)	Mean: 23.3 <18.5: 117 (5.9%) 18.5–22.9: 807 41.0%) 23–24.9: 471 (23.9%) 25–29.9: 516 (26.2%) ≥30: 56 (2.9%)	I: 1452 (73.1%) II: 163 (8.2%) III: 148 (7.5%) IV: 222 (11.2%)	Upper: 260 (13.1%) Middle: 860 (43.2%) Distal: 870 (43.7%)	Differentiated: 769 (38.6%) Undifferentiated: 1222 (61.4%)
Nam et al. [31]	Men	18,241 (61.3%)	40–49: 3128 (10.5%) 50–59: 7505 (25.2%) 60–69: 11,065 (37.2%) ≥70: 8077 (27.1%)	<18.5: 807 18.5–22.9: 7187 23–24.9: 4716 25–29.9: 5212 ≥30: 314	-	-	-
	Women	11,534 (38.7%)		<18.5: 400 18.5–22.9: 4025 23–24.9: 2936 25–29.9: 3732 ≥30: 438	-	-	-
Plazas et al. * [9]	Men	2313 (70.6%)	Mean: 65 Range: 20–89	Mean: 25 Range: 13–48	100% advanced stage	Esophagus: 258 (11.2%) Stomach: 1690 (73.1%) GEJ: 365 (15.8%)	Intestinal: 1063 (46.9%) Diffuse: 635 (26.5%) Mixed: 101 (4.4%) Unknown: 514 (22.2%)
	Women	961 (29.4%)	Mean: 63 Range: 20–89	Mean 24 Range 13–48	100% advanced stage	Esophagus: 23 (2.4%) Stomach: 862 (89.7%) GEJ: 76 (7.9%)	Intestinal: 302 (31.4%) Diffuse: 416 (43.3%) Mixed: 51 (5.3%) Unknown: 192 (20.0%)
Sah et al. [32]	Men	252 (70.4%)	Median: 59	-	-	-	-
	Women	105 (29.4%)	Median: 58	-	-	-	-
Sato et al. [34]	Men	47,535 (65.3%)	0–44: 3548 (7.5%) 45–54: 8161 (17.2%) 55–64: 13,656 (28.7%) 65–74: 13,878 (29.2%) ≥75: 8292 (17.4%)	-	Localized: 17,602 (37%) Regional: 16,378 (34.5%) Distant: 8418 (17.7%) Unknown: 5137 (10.8%)	-	Intestinal: 19,493 (41%) Diffuse: 14,362 (30.2%) Others/unknown: 13,680 (28.8%)
	Women	25,254 (34.7%)	0–44: 3406 (13.5%) 45–54: 4085 (16.2%) 55–64: 5341 (21.1%) 65–74: 6561 (26%) ≥75: 5861 (23.2%)	-	Localized: 8325 (33%) Regional: 9014 (35.7%) Distant: 4814 (19.1%) Unknown: 3101 (12.3%)	-	Intestinal: 6864 (27.2%) Diffuse: 10,401 (41.2%) Others/unknown: 7989 (31.6%)
Song et al. [37]	Men	4264 (31.8%)	Mean: 69 ± 5	-	-	Cardia: 6 (0.1%) Non-cardia: 4258 (99.9%)	-
	Women	9132 (68.2%)	Mean: 70 ± 5.7	-	-	Cardia: 0 (0%) Non-cardia: 9132 (100%)	
Song et al. [38]	Men	185 (64.5%)	Mean: 66.12 ± 9.1	-	-	-	Intestinal: 84 (45.4%) Diffuse: 89 (48.1%) Mixed: 12 (6.5%)
	Women	102 (35.5%)	Mean: 62.84 ± 11.16	-	-	-	Intestinal: 37 (36.3%) Diffuse: 58 (56.9%) Mixed: 7 (6.8%)
Suryawala et al. [42]	Men	143 (50.2%)	Mean: 69 <50: 71 ≥50: 214	-	-	-	-
	Women	142 (49.8%)		-	-	-	-
Wu et al. [43]	Men	270 (71.8%)	Mean: 65.66 ± 9.59	-	I: 61 (22.59%) II: 49 (18.15%) III: 140 (51.85%) IV: 20 (7.41%)	Lower: 152 (56.3%) Middle: 88 (32.59%) Upper: 17 (6.3%) Mixed: 13 (4.81%)	Well differentiated: 15 (5.56%) Moderately differentiated: 112 (41.48%) Poor differentiated: 143 (52.96%)
	Women	106 (28.2%)	Mean: 67.63 ± 12.59	-	I: 29 (27.36%) II: 16 (15.09%) III: 58 (54.72%) IV: 3 (2.83%)	Lower: 66 (62.26%) Middle: 35 (33.02%) Upper: 4 (3.77%) Mixed: 1 (0.94%)	Well differentiated: 5 (4.72%) Moderately differentiated: 32 (30.19%) Poor differentiated: 69 (65.09%)
Xing et al. [44]	Men	13,093 (71%)	Mean: 71.8 ± 10.1	-	Localized without LNM: 7151 (54.6%) Localized with LNM: 1389 (10.6%) Adjacent organ invasion: 1342 (10.2%) Distant metastasis: 2273 (17.4%) Unknown: 938 (7.2%)	Cardia: 1359 (10.4%) Fundus: 586 (4.5%) Body: 5803 (44.3%) Antrum: 4171 (31.9%) Unknown: 1174 (9%)	Differentiated: 8681 (66.3%) Undifferentiated: 3449 (26.3%) Unknown: 963 (7.4%)
	Women	5343 (29%)	Mean: 72.6 ± 12.5	-	Localized without LNM: 2827 (52.9%) Localized with LNM: 531 (9.9%) Adjacent organ invasion: 671 (12.6%) Distant metastasis: 903 (16.9%) Unknown: 411 (7.7%)	Cardia: 374 (7%) Fundus: 190 (3.6%) Body: 2362 (44.2%) Antrum: 1962 (36.7%) Unknown: 455 (8.5%)	Differentiated: 2802 (52.4%) Undifferentiated: 2113 (39.5%) Unknown: 428 (8%)
Yatsuya et al. * [46]	Men	46,318 (41.9%)	Mean: 58.2 40–49: 22.8% 50–59: 30.6% 60–69:31.8% 70–79: 14.9%	-	-	-	-
	Women	64,255 (58.1%)	Mean: 58.2 40–49: 22.2% 50–59: 31.6% 60–69: 32.1% 70–79: 14.2%	-	-	-	-
Yu et al. [47]	Men	83 (43.5%)	Mean: 48.9 ± 14.6 Range: 15–79	-	-	-	100% diffuse
	Women	108 (56.5%)	Mean: 43.1 ± 13.1 Range: 18–79	-	-	-	100% diffuse

* Data from Kim et al. [22] represent the average between the younger and older patient groups. Data from Maguire et al. [28] reflect the average across the Soria, Barcelona, and Mataró groups. Data from Plazas et al. [9] include patients with esophageal cancer. Data from Yatsuya et al. [46] represent the average between patients with and without a family history of gastric cancer. Abbreviations: BMI = body mass index; GEJ = gastroesophageal junction; LNM = lymph node metastasis.

## Data Availability

The original contributions presented in this study are included in the article and Supplementary Materials. Further inquiries can be directed to the corresponding author.

## Supplementary Materials

The following supporting information can be downloaded at https://www.mdpi.com/article/10.3390/jcm15124788/s1, Table S1: PRISMA 2020 for Abstracts checklist; Table S2: PRISMA 2020 checklist; Table S3: Complete search strategy for PubMed, Embase, and Web of Science databases.
