# Supplementary material for: Sex and Gender Differences in Patients with Gastric Cancer: A Systematic Review
Source: J Clin Med. 2026 Jun 19;15(12):4788. doi: 10.3390/jcm15124788 (PMC13301026; doi:10.3390/jcm15124788)
Supplement: Supplementary file 1 [file jcm-15-04788-s001.zip › Supplementary Table S3.pdf]

Supplementary Table S3: Complete search strategy for PubMed, Embase, and Web of Science databases.

Database	Search strategy	Filters applied	Records identified
PubMed	(sex based OR sex factors OR sex distribution OR sex characteristics OR sex dimorphism OR gender difference OR gender based) AND (gender[ti] OR sex[ti] OR women[ti] OR female[ti]) AND ("Stomach Neoplasms/diagnosis"[Mesh] OR "Stomach Neoplasms/epidemiology"[Mesh] OR "Stomach Neoplasms/etiology"[Mesh] OR "Stomach Neoplasms/mortality"[Mesh] OR "Stomach Neoplasms/prevention and control"[Mesh]) AND (Humans[Mesh] AND English[lang])	Humans; English	130
Embase	('gastric cancer' OR 'stomach neoplasm') AND ('sex difference' OR 'gender difference' OR female OR women) AND [humans]/lim AND [english]/lim	Humans; English	165
Web of Science	(sex difference OR gender difference OR sex factor OR female OR woman OR gender OR sex) AND (gastric cancer OR gastric neoplasm OR stomach cancer OR stomach neoplasm) AND english AND humans	Article document type; English language; Human Medicine Medical Sciences; Oncology; Public Health; Geriatrics; Molecular Genetics; Medical Sciences	28

Searches were conducted in PubMed, Embase, and Web of Science up to February 2026. Reference lists of relevant systematic reviews and meta-analyses were also manually screened to identify additional eligible studies
